# Supplementary material for: Human orbitofrontal cortex signals decision outcomes to sensory cortex during behavioral adaptations
Source: Nat Commun. 2023 Jun 15;14:3552. doi: 10.1038/s41467-023-38671-7 (PMC10272188; doi:10.1038/s41467-023-38671-7)
Supplement: Supplementary file 3 — Reporting Summary [file 41467_2023_38671_MOESM3_ESM.pdf]

nature portfolio

Corresponding author(s): Abhishek Banerjee and Burkhard Pleger

Last updated by author(s): Apr 5, 2023

Reporting Summary

Nature Portfolio wishes to improve the reproducibility of the work that we publish. This form provides structure for consistency and transparency in reporting. For further information on Nature Portfolio policies, see our Editorial Policies and the Editorial Policy Checklist.

Statistics

For all statistical analyses, confirm that the following items are present in the figure legend, table legend, main text, or Methods section.

n/a	Confirmed	
☐	☒	The exact sample size (*n*) for each experimental group/condition, given as a discrete number and unit of measurement
☐	☒	A statement on whether measurements were taken from distinct samples or whether the same sample was measured repeatedly
☐	☒	The statistical test(s) used AND whether they are one- or two-sided<br>*Only common tests should be described solely by name; describe more complex techniques in the Methods section.*
☐	☒	A description of all covariates tested
☐	☒	A description of any assumptions or corrections, such as tests of normality and adjustment for multiple comparisons
☐	☒	A full description of the statistical parameters including central tendency (e.g. means) or other basic estimates (e.g. regression coefficient) AND variation (e.g. standard deviation) or associated estimates of uncertainty (e.g. confidence intervals)
☐	☒	For null hypothesis testing, the test statistic (e.g. *F*, *t*, *r*) with confidence intervals, effect sizes, degrees of freedom and *P* value noted<br>*Give P values as exact values whenever suitable.*
☒	☐	For Bayesian analysis, information on the choice of priors and Markov chain Monte Carlo settings
☒	☐	For hierarchical and complex designs, identification of the appropriate level for tests and full reporting of outcomes
☐	☒	Estimates of effect sizes (e.g. Cohen's *d*, Pearson's *r*), indicating how they were calculated

Our web collection on statistics for biologists contains articles on many of the points above.

Software and code

Policy information about availability of computer code

Data collection	Behavioral performance was recorded using Presentation software (version 20.1, Neurobehavioral Systems, 371 Berkeley, CA, USA) through LumiTouch keypads (Photon Control Inc., Burnaby, BC, Canada). fMRI data was collected using a multi-band echo-planar imaging (EPI) sequence with a multi-band acceleration factor of 2 on a Philips Achieva 3.0 T scanner using a 32-channel head coil.
Data analysis	Behavioral data was analysed in Matlab R2017b using custom codes. The modeling of behavioral data was performed using a two-level version of the HGF toolbox (v7.0, https://www.tnu.ethz.ch/de/software/tapas.html). The preprocessing and GLM analysis of fMRI data was performed using SPM12 (Wellcome Department of Imaging Neuroscience, University College London, UK; http://www.fil.ion.ucl.ac.uk/spm) implemented in Matlab R2017b (MathWorks Inc). The representational similarity analysis of fMRI data was implemented using the RSA toolbox available on GithHub (V1.0, https://github.com/rsagroup/rsatoolbox) and custom codes developed in Matlab R2017b. The psychophysiology interaction analysis of fMRI data was performed using the generalized PPI (gPPI) toolbox (V13.1, https://www.nitrc.org/projects/gppi). The fMRI results were presented using MRIcron (https://www.nitrc.org/projects/mricron). The required sample size was estimated using G*Power (version 3.1.9.2, https://www.psychologie.hhu.de/arbeitsgruppen/allgemeine-psychologie-und-arbeitspsychologie/gpower). The custom codes that were applied to assess the findings of this study were deposited at https://github.com/Bin-A-Wang/ReversalLearning_lOFC_S1, and Zenodo repository with a DOI (https://doi.org/10.5281/zenodo.7805722).

For manuscripts utilizing custom algorithms or software that are central to the research but not yet described in published literature, software must be made available to editors and reviewers. We strongly encourage code deposition in a community repository (e.g. GitHub). See the Nature Portfolio guidelines for submitting code & software for further information.

Data

Policy information about availability of data

All manuscripts must include a data availability statement. This statement should provide the following information, where applicable:

- Accession codes, unique identifiers, or web links for publicly available datasets
- A description of any restrictions on data availability
- For clinical datasets or third party data, please ensure that the statement adheres to our policy

The behavioral data and processed fMRI data has been deposited at Sciebo (https://ruhr-uni-bochum.sciebo.de/s/m5Wi0XavNLQ05hT) and is publicly available as of the date of publication. The raw imaging data is not publicly available due to restrictions related to the individual information that could compromise the privacy of research participants. Source data are provided with this paper.

Human research participants

Policy information about studies involving human research participants and Sex and Gender in Research.

Reporting on sex and gender	The sex was assessed to match female and male participants in our study. The sex was determined by participants' self-report. The information to disaggregate gender data has not been collected. No gender/sex-based analyses were performed because gender/sex is not the factor we focused in our study.
Population characteristics	Female and male participants (mean age ± SD: 24.5 ± 3.3 years) were recruited. All participants were right-handed and had normal or corrected to normal vision. Participants with a history of psychiatric or neurological disorders as well as any those taking regular medication or presented contradictions to MRI scanning (i.e., metal implants, pregnancy, breast feeding, claustrophobia) were excluded.
Recruitment	We posted our advertisement on the virtual blackboard of the Ruhr-University Bochum. All participants who meet the criterion mentioned above were included. There was no self-selection bias.
Ethics oversight	The study was approved by the local ethics committee of the medical faculty at the Ruhr-University Bochum.

Note that full information on the approval of the study protocol must also be provided in the manuscript.

Field-specific reporting

Please select the one below that is the best fit for your research. If you are not sure, read the appropriate sections before making your selection.

☒ Life sciences ☐ Behavioural & social sciences ☐ Ecological, evolutionary & environmental sciences

For a reference copy of the document with all sections, see nature.com/documents/nr-reporting-summary-flat.pdf

Life sciences study design

All studies must disclose on these points even when the disclosure is negative.

Sample size	The sample size was assessed with the free-source software G*Power 3.1.9.2. The required sample size was calculated using the two-tailed t-test between two dependent samples. Due to our previous study (Wang and Pleger, 2020, Cerebral Cortex), we expected an high effect size at 0.7. Error probability was set to 0.05. The predicted sample size was 29. Considering possible exclusions of participants, we recruited 40 participants in total.
Data exclusions	Participants who failed during the training session were excluded from fMRI scanning because they were unable to correctly identify the tactile patterns. Participants who performed the task during fMRI scanning but failed to learn the correct associations between tactile stimuli and responses were excluded from further data analysis.
Replication	1. The switch-related lOFC signals, reported in the submitted manuscript, are a replication of an earlier finding identified in a different human study sample. In this previous study, participants also presented significant lOFC activation when they had to adapt their decision strategy from trial to trial (Wang and Pleger, 2020, Cerebral Cortex).<br>2. The main finding of the current study is that interactions between OFC and S1, as observed in mice enganged in the same task as the human participants in the present study (Banerjee et al 2020, Nature), can be fully replicated.
Randomization	Our study consisted only of one experimental group. Randomization was not applicable.
Blinding	No blinding was performed as there was no group allocation in this study.

Reporting for specific materials, systems and methods

We require information from authors about some types of materials, experimental systems and methods used in many studies. Here, indicate whether each material, system or method listed is relevant to your study. If you are not sure if a list item applies to your research, read the appropriate section before selecting a response.

Materials & experimental systems

n/a	Involved in the study
☒	☐ Antibodies
☒	☐ Eukaryotic cell lines
☒	☐ Palaeontology and archaeology
☒	☐ Animals and other organisms
☒	☐ Clinical data
☒	☐ Dual use research of concern

Methods

n/a	Involved in the study
☒	☐ ChIP-seq
☒	☐ Flow cytometry
☐	☒ MRI-based neuroimaging

Magnetic resonance imaging

Experimental design

Design type: Event-related task design

Design specifications: The fMRI experiment consisted of 540 trials in total, which were split into 3 runs. Each run lasted ~16 mins and consisted of 4 blocks. Each block contained 45 trials and each trial's duration was ~3 seconds. Trials were presented with randomized intertrial intervals ranging between 1500 and 3000ms, in 100ms steps.

Behavioral performance measures: Behavioral responses (i.e., Go or No-Go) were recorded, and the percentage of correct responses at different stages of the task were used to assess whether participants performed as expected

Acquisition

Imaging type(s): Functional MRI

Field strength: 3 Tesla

Sequence & imaging parameters: Functional scans were collected using a multi-band echo-planar imaging (EPI) sequence with a multi-band acceleration factor of 2. Thirty eight transaxial slices parallel to the anterior-posterior commissure (AC-PC) covering the whole brain were acquired with a voxel size of 2 x 2 x 3 mm3, TR = 2,200 ms, TE = 24 ms, flip angle =90, the field of view 224 mm, and no interslice gap. High-resolution T1-weighted structural images were also acquired using isotropic T1 TFE sequence (176 transversally oriented slices, voxel size: 1 x 1 x1 mm3, field of view 240 x 176 mm2).

Area of acquisition: whole brain scan

Diffusion MRI ☐ Used ☒ Not used

Preprocessing

Preprocessing software: The functional scans were preprocessed using the Statistical Parametric Mapping software SPM12 (Wellcome Department of Imaging Neuroscience, University College London, UK; http://www.fil.ion.ucl.ac.uk/spm) implemented in Matlab R2017b (MathWorks Inc).

Normalization: The individual T1w image was normalized to the Montreal Neurological Institute (MNI) reference space using the unified segmentation approach and parameters were next applied to the BOLD-sensitive EPI scans.

Normalization template: MNI-space standard templates in SPM was used for normalization

Noise and artifact removal: Data were high pass filtered at 1/128 Hz to remove low-frequency signal drifts. Motion-correction parameters were included into the GLM, which allows movements effects to be discounted when looking for brain activations

Volume censoring: The first five volumes in each run were removed

Statistical modeling & inference

Model type and settings: The first GLM, used to analyze the univariate BOLD effect, included four regressors of interest per block (LN, LE, RN, RE). The onset of events within these 4 regressors were locked to the onset of the outcome in each trial. For each of these regressors, the absolute value of trial-by-trial outcome prediction error derived from HGF model was defined as a parametric modulator. Two additional regressors were included to account for the onset of outcomes presentation (correct or incorrect). The onset

of events within these 6 regressors were locked to the onset of the outcome in each trial. Two additional regressors of no interest accounted for the presentation of the stimuli (all trials collapsed to a single regressor, time-locked to the onset of cue presentation) and invalid trials (i.e., late responses). For the first level of univariate GLM analysis, two contrasts ("RN > LE" and "RE > LE") were assessed to reveal changes of BOLD responses after the reversal.
The second GLM, used to assess the representational similarity between different phases of learning using RSA, consisted of the unsmoothed fMRI data separated into 16 regressors of interest per block. These 16 regressors accounted for trials of the four different phases of the task (LN, LE, RN, RE), divided into the different outcomes (HIT, Correct Rejection or CR, False Alarm or FA, MISS).
The third GLM, applied to assess functional connectivity using PPI, included five regressors of interest, consisting of physiological, psychological and PPI regressors. The physiological regressor was defined as the fMRI time-series extracted from a seed region. Two psychological regressors accounted for trials before and after the reversal (i.e., LE&RN or LE&RE). Two PPI regressors accounted for the interactions between the physiological variable and psychological regressors by extracting and deconvolving the time-series from the seed region, multiplying it by the psychological regressor and then convolving the output with the hemodynamic response function. To account for additional unwanted variance, the regressors of no interest included in the first GLM described above were also included, including two regressor accounted for outcomes (correct or incorrect trial), and two regressors representing the presentation of the stimuli and invalid trials.

Effect(s) tested

For the univariate GLM analysis, the fMRI BOLD signal during Reversal Naïve (RN) trials were first contrasted with the fMRI BOLD signal during Learning Expert (LE) trials to measure the BOLD response to the immediate effect of the rule switch. Second, to measure the BOLD response to the adaptation after re-learning, Reversal Expert (RE) trials were contrasted with Learning Expert (LE) trials.The contrast images were next applied to the second-level one-sample t-test to reveal group results (two-sided, thresholded at p=0.05, small volume FWE corrected).
For RSA analysis, the similarity effects were tested at the group level using two approaches: (1) one-sided Wilcoxon signed-rank test across participants; (2) one-sided permutation test where the null distribution was generated by estimating the group average 10,000 times, after permuting the identity of trials in the RDM on each iteration
For PPI analysis, the first-level contrast images were created using the PPI regressor of the interaction between the physiological variable and LE trials, as well as the interaction between the physiological variable and RE trials. The contrast images (i.e., RN > LE and RE > LE) were next applied to the group-level one-sample t-test (two-sided). Small volume correction was used by restricting the search volume to either the OFC or the S1 mask. All PPI analyses were thresholded with SVC for multiple comparison at FWE-corrected peak-level of $p < 0.05$

Specify type of analysis: ☐ Whole brain ☒ ROI-based ☐ Both

Anatomical location(s): SPM Anatomy Toolbox

Statistic type for inference (See Eklund et al. 2016): voxel-wise

Correction: small-volume FWE peak-level correction at $p < 0.05$

Models & analysis

n/a	Involved in the study
☐	☒ Functional and/or effective connectivity
☒	☐ Graph analysis
☐	☒ Multivariate modeling or predictive analysis

Functional and/or effective connectivity: Regression coefficient of the psycho-physiological Interaction (PPI) term

Multivariate modeling and predictive analysis: Independent variables were voxel-wise fMRI responses to outcomes; The response pattern to outcomes in S1 and lateral OFC during initial learning (LE) and after the reversal (RN, RE) were compared using Pearson's correlations to establish a cross-phases representational dissimilarity matrix (RDM) for representational similarity analysis (RSA).
